# Famous Poisoners in Fiction

**Published:** 1894-07-21

**Authors:** 


### Famous Poisoners in Fiction.

### X.—THE FAVOURITE POISONS OF AUTHORS AND DRAMATISTS.

### Part II.

Few dramatists have been happy in their descriptions of death by poison. Cleopatra, oftimes as she has died in fiction, has never yet died as she must have in reality, if indeed, with the poison of an asp (and the supposition has strong historical grounds) she, for love of handsome Anthony, rid herself of this frail web called life. It is scarcely likely she felt the "peace, peace," Shakespeare puts into her dying lips followed by the lines—

> Dost thou not see my baby at my breast
> That sucks the nurse asleep,
> As sweet as balm, as soft as air, as gentle.

But the Singing Swan of Avon is happier in his description of the death of the King of Denmark by his brother's hand. The "juice of cursed herbenon," with which Hamlet's father was poisoned, and which

> Holds such an enmity with blood of man
> That swift as quicksilver it courses thro'
> The natural gates and alleys of the body,
> And with a sudden rigour it doth posset
> And curd like aigre droppings into milk
> The thin and wholesome blood, is supposed to have been the well-known medicine and narcotic, herbane, an overdose of which is death.

The belief that poison could be introduced into the system by being poured into the ear, was shared by the medical profession of that day, and is most likely true. Ambrose Paré, the French chirurgeon, who lived about the same time as the great dramatist, was suspected of having administered poison in this manner to Francis II. of France. Indeed, there seems scarcely any doubt that he died from the effects of posion so administered. Nathaniel Lee, in his "Alexander the Great," is particularly unfortunate in his description of the effects of poison. Alexander drinks of a poison which is written about as of "such exalted force" that,

> Mixed with his wine a single drop gives death,
> And sends him howling to the shades beneath.

Yet he, after partaking of this poison, lives through the whole of one act and the greater part of the second, raves with delirium, regains his senses, and finally dies after making a very eloquent speech. Never had poison so strange an effect!

In Philip Massinger's play, "The Duke of Milan," the villain of the piece, Francisco, dusts over a plant with a poisonous powder. Eugenia is holding this plant when Ludovico Sporza kisses her hand, and from this slight contact he dies with remarkable rapidity. The whole scene is obviously absurd, for there is no poison so strong as to kill a man in such a manner, not even aconite, the most vicious poison known, and which, in all its intensity, has only been discovered of comparatively recent years.

But there is at the present day a poison the effects of which are so extraordinary that it proves again the old adage that truth is stranger than fiction. After it has been swallowed, the person "walks about for an interval of time varying from a quarter of an hour to two hours, his skin becomes of a strange purplish shade, but he may still feel quite well; then suddenly the fatal symptoms set in with appalling rapidity, and in a few seconds the victim is dead." "For anyone who delights in constructing stories of sensation," writes Mr. Wynter Blyth in his pamphlet on "Poison Lore," "these, the occasional effects of nitro-benzene, afford considerable, although, perhaps, not commendable scope." Cantharides, an animal poison, the same as the Spanish fly, has sometimes been made use of in fiction. This was a very common poison in the middle ages, being oftimes mixed with pepper, which it greatly resembled. The life of Sir Thomas Overbury is supposed to have been attempted in this manner.

One of the strangest poisons ever introduced into the pages of fiction is that written of by James Payn in his novel "Halves." Again it is the old, old story, the love of money, which induces the crime; buc Mrs. Raeburn's method of poisoning is original in the extreme. Who would dream that death lurks even in our innocent-looking drawing-room furniture? Very finely chopped horsehair is her novel weapon for removing her niece from this weary world. By its use she brings on an illness which puzzles completely the doctor, but by one of those convenient accidents, so very common in three-volumed novels, the would-be murderess was seen pulling the horsehair out of the padded sofa. Suspicion was thus aroused, and the pepper used to flavour Gertrude Floyd's dinner on being examined was found to be too large in size, and too concentrated also in essence.

Antidotes are largely employed as life-saving machinery in novels; this is mainly due to the reader's usual preference for books which end happily, and with the traditional wedding bells. It is not, however, the fact of the antidote itself, for nearly every poison has its especial antidote, but the convenient fact that the antidote is always ready at hand when needed, which forms the subtle difference between the world of romance and that prosaic one of reality. In both these worlds the terrible lesson of the awful depravity of the heart of man is taught most positively, for in both fellow-men and fellow-women use against each other the stealthy and deadly weapon of poison, until one begins to wonder why the fruits of the earth should contain not only the power of sustaining life, but also the more awful one of dealing forth death. Were the opportunities less frequent, surely the poisoner's weapon would be less frequently used.

And yet in almost every case the deadly poison is also a valuable and useful medicine, and thus it can become either a means of life unto life or of death unto death. Truly

> There is a soul of goodness in things evil,
> Would man observingly distil it out.

One reads that the Comte de Monte Christo, when he and Madame de Villefort were talking of well-known poisons and poisoners, of Mithridatus, who breakfasted every morning with a cup of poison *à la crème*, of Baron de Trench of Desmes, and of the infamous Borgias, he congratulated Madame de Villefort on her knowledge of the science of poison; but may it not be as well that it is a lore little known and little popular in England, however much it may be studied amongst Oriental nations, who believe still in this powerful, successful, if mean, manner of disposing of an inconvenient friend or troublesome foe. It would be a rueful day for our country if ever toxicology was added to the many 'ologies the Board school children learn, for the holiest affections sometimes inspire also the most terrible passions, as in all characters good and evil are intermingled strangely. It is strange how often murders come to light; how extraordinarily careless the perpetrators are; how the poisoner seems always to leave behind him some damning evidence of his guilt. It may be as the Comte de Monte Christo is supposed to point out, that the cause of the bunglings, which so often follow the poisoner's stupid attempts at murder, is the result of the utter want of medical science which makes our playwrights and authors represent the effect of poisons, as illustrated on the stage, as far from the real actual truth as possible, as well as to omit in nearly every case the consequences which, in real life, the awkward and foolish poisoner is bound to bring down on his unfortunate head. But another and more true reason is that while the stupid poisoner is detected, and folk censoriously quote the proverb, "Murder will out," the clever, cunning nineteenth century Elisirs and Medicis keep their dark secrets unrevealed, unguessed at, whilst year by year the dustheap of undiscovered crime grows larger and larger, until it assumes the proportions of a mountain, the actual height of which is known alone to God's recording angel.

